# Development and validation of machine learning models for predicting no. 253 lymph node metastasis in left-sided colorectal cancer using clinical and CT-based radiomic features

**DOI:** 10.1186/s40644-025-00876-y

**Published:** 2025-04-29

**Authors:** Hongwei Zhang, Kexin Wang, Shurong Liu, Guowei Chen, Yong Jiang, Yingchao Wu, Xiaocong Pang, Xiaoying Wang, Junling Zhang, Xin Wang

**Affiliations:** 1 Department of Gastrointestinal Surgery, Peking University First Hospital, No. 8, Xishiku Street, Xicheng District, Beijing, 100034 China; 2 School of Basic Medical Sciences, Capital Medical University, No. 10, Xitoutiao, Youanmenwai Street, Fengtai District, Beijing, 100069 China; 3 Department of Pharmacy, Peking University First Hospital, No. 8, Xishiku Street, Xicheng District, Beijing, 100034 China; 4 Department of Radiology, Peking University First Hospital, No. 8, Xishiku Street, Xicheng District, Beijing, 100034 China

**Keywords:** Colorectal cancer, Lymph node metastasis, Radiomics, Machine learning, Prediction model

## Abstract

**Background:**

The appropriate ligation level of the inferior mesenteric artery (IMA) in left-sided colorectal cancer (CRC) surgery is debated, with metastasis in No. 253 lymph node (No. 253 LN) being a key determining factor. This study aimed to develop a machine learning model for predicting metastasis in No. 253 LN.

**Methods:**

We retrospectively collected clinical data from 2,118 patients with left-sided CRC and contrast-enhanced CT images from 310 of these patients. From this data, a test set, a training set, and a temporal validation set were constructed. Logistic regression models were used to develop a clinical model, a CT model, and a radiomics model, which were then integrated into a combined model using logical rules. Finally, these models were evaluated using metrics such as the area under the receiver operating characteristic curve (AUC), precision-recall (PR) curves, decision curve analysis (DCA), net reclassification improvement (NRI), and integrated discrimination improvement (IDI).

**Results:**

A clinical model, a CT model, and a radiomics model were constructed using univariate logistic regression. A combined model was developed by integrating the clinical, CT, and radiomics models, with positivity defined as all three models being positive at a 90% sensitivity threshold. The clinical model included six predictive factors: tumor site, endoscopic obstruction, CEA levels, growth type, differentiation grade, and pathological classification. The CT model utilized largest lymph node average CT value, short-axis diameter and long-axis diameter. The radiomics model incorporated maximum gray level intensity within the region of interest, large area high gray level emphasis, small area high gray level emphasis and surface area to volume ratio. In the test set, the AUCs for the clinical, CT, radiomics, and combined models were 0.694, 0.663, 0.72, and 0.663, respectively, while in the temporal validation set, they were 0.743, 0.629, 0.716, and 0.8. Specifically, the combined model demonstrated a sensitivity of 0.8 and a specificity of 0.8 in the temporal validation set. By comparing the PR and DCA curves, the combined model demonstrated better performance. Additionally, the combined model showed moderate improvements in INR and IDI compared to other models.

**Conclusion:**

A clinical and CT-based radiomics model shows promise in predicting No. 253 LN metastasis in left-sided CRC and provides insights for optimizing IMA ligation strategies.

**Supplementary Information:**

The online version contains supplementary material available at 10.1186/s40644-025-00876-y.

## Background

Colorectal cancer (CRC) accounts for nearly 10% of all cancer incidences and deaths, ranking third in incidence and second in mortality [1]. Surgical treatment remains a pivotal treatment for colorectal cancer, particularly in localized cases. The optimal level of the inferior mesenteric artery (IMA) ligation during left-sided CRC surgery is still debated [2, 3, 4]. One approach is high ligation at the root of IMA, which simplifies the procedure and enables comprehensive dissection of No. 253 lymph node (No. 253 LN). Conversely, low ligation at the distal branch of the left colic artery (LCA) preserves better blood supply to the proximal anastomosis, improving urogenital function preservation but carries a higher risk of leaving residual lymph nodes [5, 6]. The American Society of Colon and Rectal Surgeons Clinical Practice Guidelines recommend high ligation only for patients at high risk of No. 253 LN metastasis [7].

A significant challenge in this strategy is accurately determining metastasis in No. 253 LN. Radiological assessments have limited accuracy, with computed tomography (CT) imaging sensitivity ranging from 28.1 to 52.4% and specificity from 58.8 to 99.2%. Magnetic resonance imaging (MRI) offers slightly better sensitivity (36.0–63.1%) but similar specificity (51.9–83.0%) [8]. However, MRI is seldom performed on patients with tumors outside the lower rectum [9]. Intraoperative assessments by surgeons also lack precision, with an area under the curve (AUC) of 0.509 [10]. Recently, several studies have utilized clinical data to develop predictive models, enhancing the ability to predict metastasis in No. 253 LN [10, 11].

Radiomics, which involves high-throughput extraction of quantitative features from medical images, has been effectively used in the preoperative staging of CRC patients [12, 13, 14, 15]. However, to the best of our knowledge, there is currently no radiomics predictive model for No. 253 LN metastasis. The potential of information from CT, commonly used in CRC assessment, remains underexplored. This study aims to utilize radiomic features derived from CT, combined with clinical characteristics, to develop a machine learning model for predicting No. 253 LN metastasis. This model is expected to provide valuable guidance in determining the optimal IMA ligation strategy.

## Materials and methods

### Patients’ selection

We retrospectively collected cases of patients who underwent surgical treatment for left-sided CRC at Peking University First Hospital from July 1, 2009, to December 31, 2023. Inclusion criteria were: (1) Radical surgery for left-sided CRC at our hospital; (2) Intraoperative dissection of No. 253 LN with separate pathological diagnosis; (3) Complete clinical data, including basic information, surgical records, and pathology reports. Exclusion criteria were: (1) History of other malignant tumors; (2) Incomplete clinical data. We further refined the cohort to establish a radiomics cohort. Inclusion required preoperative abdominal enhanced CT scans within one month at our hospital, excluding those who received neoadjuvant therapy. Given the low incidence of positive No. 253 LN metastasis, we used a 1:2 positive-to-negative propensity score matching (R package MatchIt) with a caliper of 0.03 for patients from July 1, 2009, to June 30, 2022, to form training and testing sets. The training and testing sets were randomly allocated in a ratio of 7:3. For patients from July 1, 2022, to December 31, 2023, a 1:2 random matching was used to create a temporal validation set. This study was approved by the ethics committee of the Peking University First Hospital (ID: 2023-414-002) with waived written informed consent.

### Lymph node annotation

**Fig. 1:**
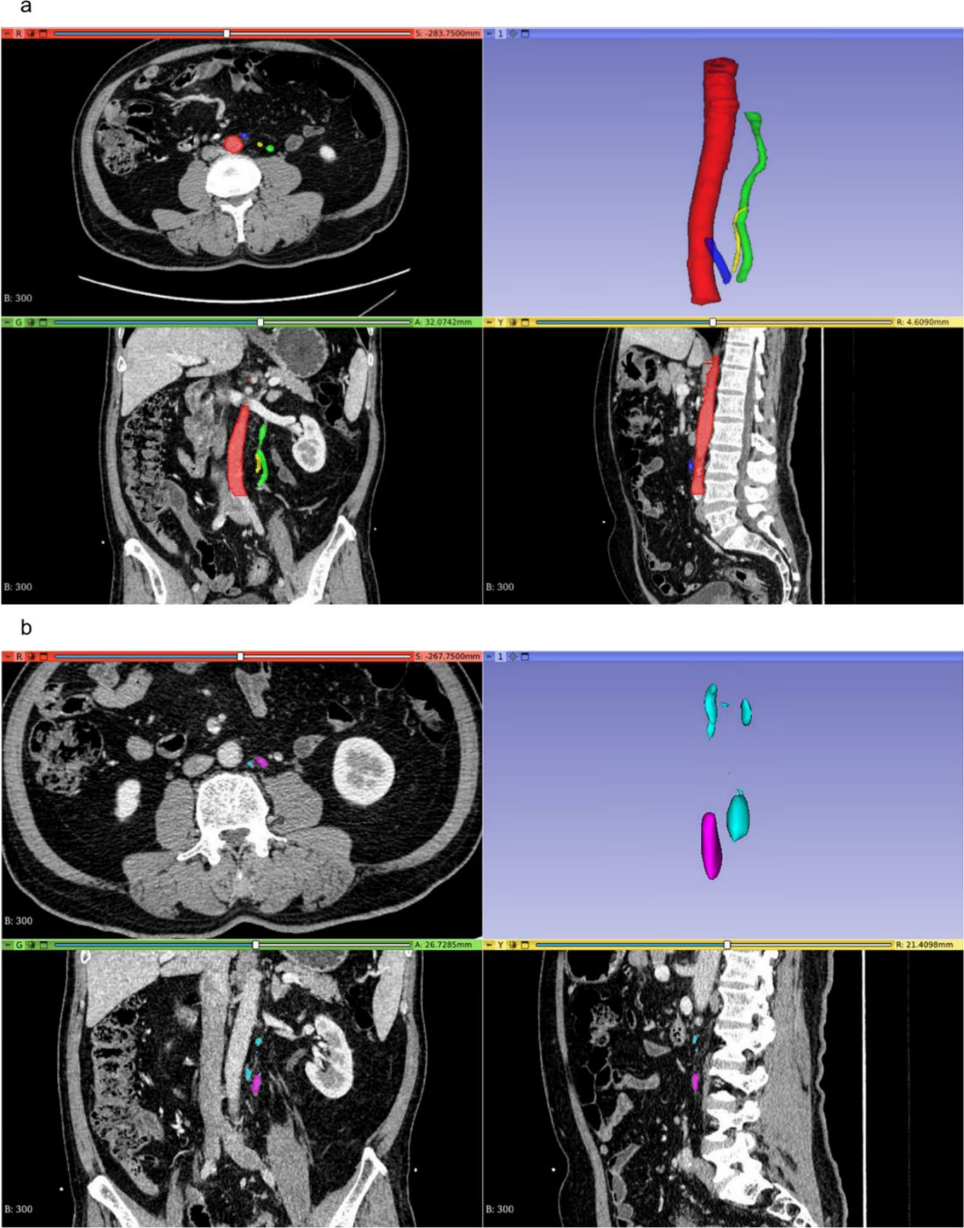
The annotation of No. 253 LN region on CT images (**a**). The four views represent axial (top left), coronal (bottom left), sagittal (bottom right), and 3D (top right) images. The red area represents the aorta, the green area represents IMV, the blue area represents IMA, and the yellow area represents LCA. The upper boundary for annotating No. 253 LN was the starting point of IMA, with the right boundary being the left edge of the aorta, the left boundary being the right edge of IMV, and the lower boundary extending from IMA to the intersection of LCA and IMV. The annotation of all the lymph nodes in No. 253 LN region (**b**). The purple area represents the largest lymph node and the light blue area represents other lymph nodes in No. 253 LN region. LN = lymph node; IMV = inferior mesenteric vein; IMA = inferior mesenteric artery; LCA = left colic artery

Two radiologists annotated all the lymph nodes in the No. 253 region use 3D Slicer image computing platform provided by Slicer Community (https://www.slicer.org/) [16]. Initially, a junior radiology resident with three years of experience performed the annotations. Subsequently, a senior abdominal imaging specialist with 30 years of experience reviewed and revised the annotations. The No. 253 LN refer to the mesenteric lymph nodes along the root of IMA extending from the origin of IMA to the origin of LCA. These nodes represent the third station of lymph nodes for metastasis in left-sided CRC patients. The upper boundary for annotating No. 253 LN was the origin of IMA, with the right margin along the left edge of the aorta, the left margin along the right edge of the inferior mesentery vein (IMV), and the lower boundary from the continuation of IMA to the point where LCA intersects with IMV. Initially, the radiologists annotated all the lymph nodes in this region, followed by the selection of the largest lymph node based on this annotation. Figure 1 provides an illustrative example of annotated lymph nodes.

### Feature extraction

For the clinical model, patients’ demographic data, comorbidities, examination results, surgical details, and pathological findings were collected. For the CT model, all No. 253 LN and the largest lymph node were measured, included volume, CT value, short-axis diameter (SAD) and long-axis diameter (LAD). Due to the difficulty in achieving a one-to-one correspondence between CT images and pathology, we selected the largest lymph node most likely to exhibit metastasis for radiomics feature extraction. For the radiomics model, PyRadiomics software package (https://pyradiomics.readthedocs.io/en/latest/) [17] was used to extract features using the largest lymph node as the region of interest (ROI). This involved extracting 102 image features for each ROI, including 14 shape-based features, 18 first-order features, 24 Gy-level co-occurrence matrix (GLCM) features, 14 Gy-level dependence matrix (GLDM) features, 16 Gy-level run length matrix (GLRLM) features and 16 Gy-level size zone matrix (GLSZM) features.

### Model development

Patients with CT images were randomly divided into a training set (*n* = 187) and a test set (*n* = 78) in a 7:3 ratio. Those without CT images were included in the training set (*n* = 1995) to develop a clinical model. The CT and radiomics models were trained using this training set. Then, a combined model was developed by integrating the clinical, CT, and radiomics models, with positivity defined as all three models being positive at a 90% sensitivity threshold. The performance of all four models was evaluated in both the test set and temporal validation set. All categorical variables included in the model were converted into dummy variables, while continuous variables were normalized using mean normalization.

### Clinical model

Using clinical information as predictive factors, the clinical model initially employed a univariate logistic regression model. Each clinical indicator was treated as an independent variable, and associations with the occurrence of the target event of No. 253 LN metastasis were established to observe the impact of each indicator on the target event. Subsequently, a multivariate logistic regression model was constructed, and multiple clinical indicators were considered to comprehensively analyze their joint influence on the target event. The stepwise Akaike information criterion (AIC) method (R package MASS) was used for feature selection. Next, we calculated the variance inflation factor (VIF) of the model using the R package car and removed the variables that exhibited multicollinearity. The selection of final predictive factors for the multivariable logistic regression was based on the results of univariable logistic regression and the clinical significance of the variables.

### CT model

Following a similar approach to fitting the clinical model, the CT model used CT evaluation indices as predictive factors to obtain univariate logistic regression and multivariate regression models. Additionally, variables exhibiting multicollinearity were removed by calculating the VIF.

### Radiomics model

The radiomics model was also developed using a logistic regression framework. Specifically, to prevent multicollinearity among variables, the Pearson correlation analysis was conducted to assess pairwise correlations between different features. If the Pearson correlation coefficient (PCC) between two features was greater than 0.99, one of the features was randomly removed. The selection of final predictive factors was based on clinical significance and verified through the examination of VIF.

### Combined model

**Fig. 2:**
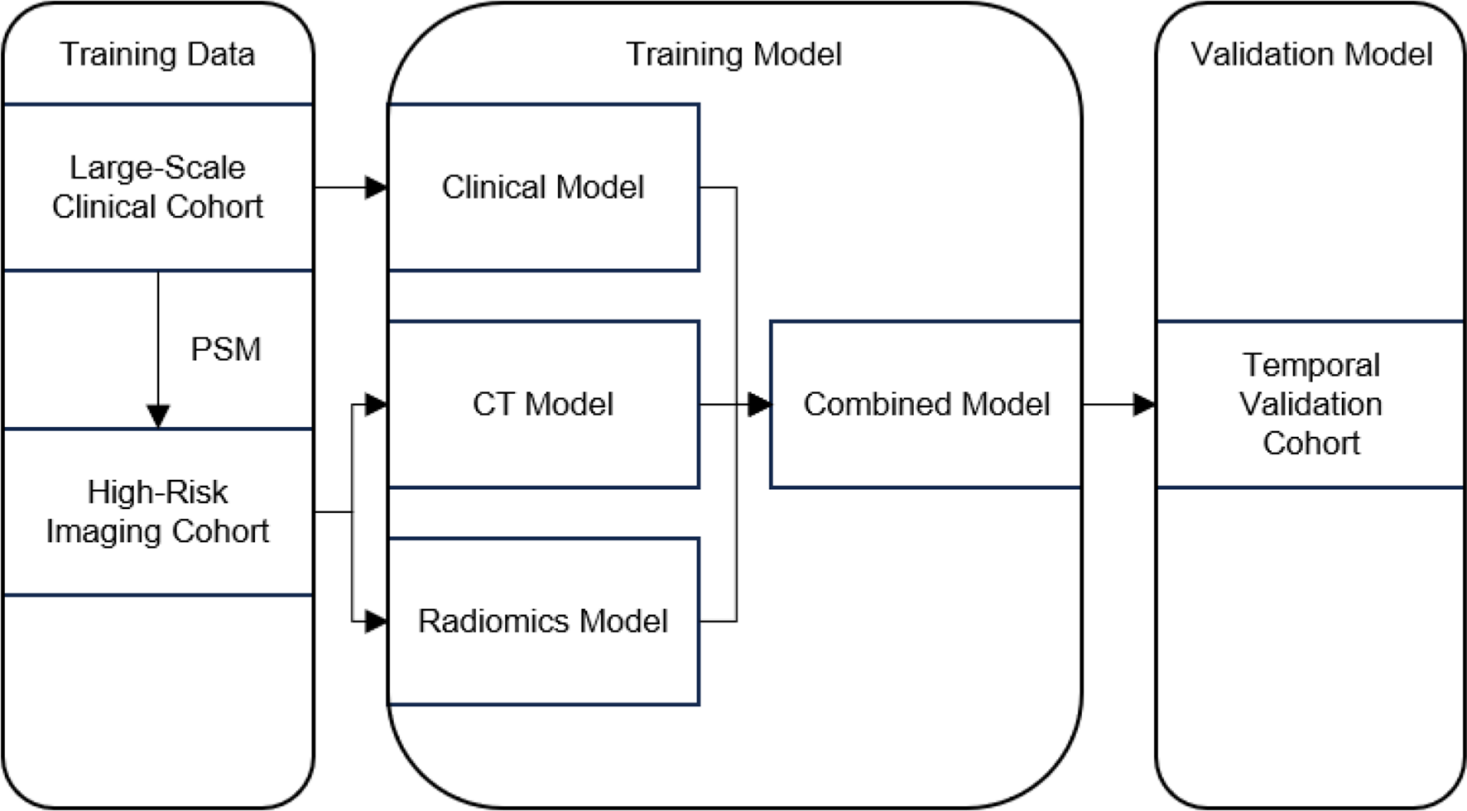
The construction methodology of clinical, CT, Radiomics, and combined model. A large-scale clinical cohort was used to train the clinical model, while imaging data were collected from high-risk individuals identified through propensity score matching based on clinical information to construct an imaging cohort for training the CT and radiomics models. Then, a clinical model, a CT model, and a radiomics model were constructed using univariate logistic regression. Subsequently, a combined model was developed by integrating the clinical, CT, and radiomics models, with positivity defined as all three models being positive at a 90% sensitivity threshold. Finally, the models were evaluated using a temporal validation cohort. PSM = propensity score matching

The combined model was created by integrating the clinical, CT, and radiomics models, with each individual model set to a threshold that achieved 90% sensitivity. The combined model was considered positive only when all three individual models indicated a positive result. The construction methodology of aforementioned four models is summarized in Fig. 2.

### Model evaluation

Using the four models, the likelihood of No. 253 LN metastases was predicted for each patient in the test set, and the predicted probability was obtained for each sample. The performance of the models was assessed using receiver operating characteristic (ROC) curves to compare the AUC of the different models. Further comparisons among different models were conducted in the test set using precision–recall (PR) curves and decision curve analysis (DCA). The net reclassification improvement (NRI) and integrated discrimination improvement (IDI) of the combined model relative to the other three models were calculated (R package PredictABEL). The cutoff value for NRI was determined using the threshold that maximizes the Youden index.

### Statistical analyses

Statistical analysis was performed using R 4.4.1 software. The normality of the numeric data was assessed using the Kolmogorov‒Smirnov test. Numeric data conforming to a normal distribution are presented as the mean ± standard deviation, while non-normally distributed numeric data are expressed as the median [interquartile range]. Categorical data are presented as numbers (%). The comparison of numeric data utilized either the t test or Mann‒Whitney U test. For the comparison of categorical data, the chi-square test or Fisher’s exact probability test was used. Differences were considered statistically significant at *P* < 0.05.

## Results

### Demographic information

**Fig. 3:**
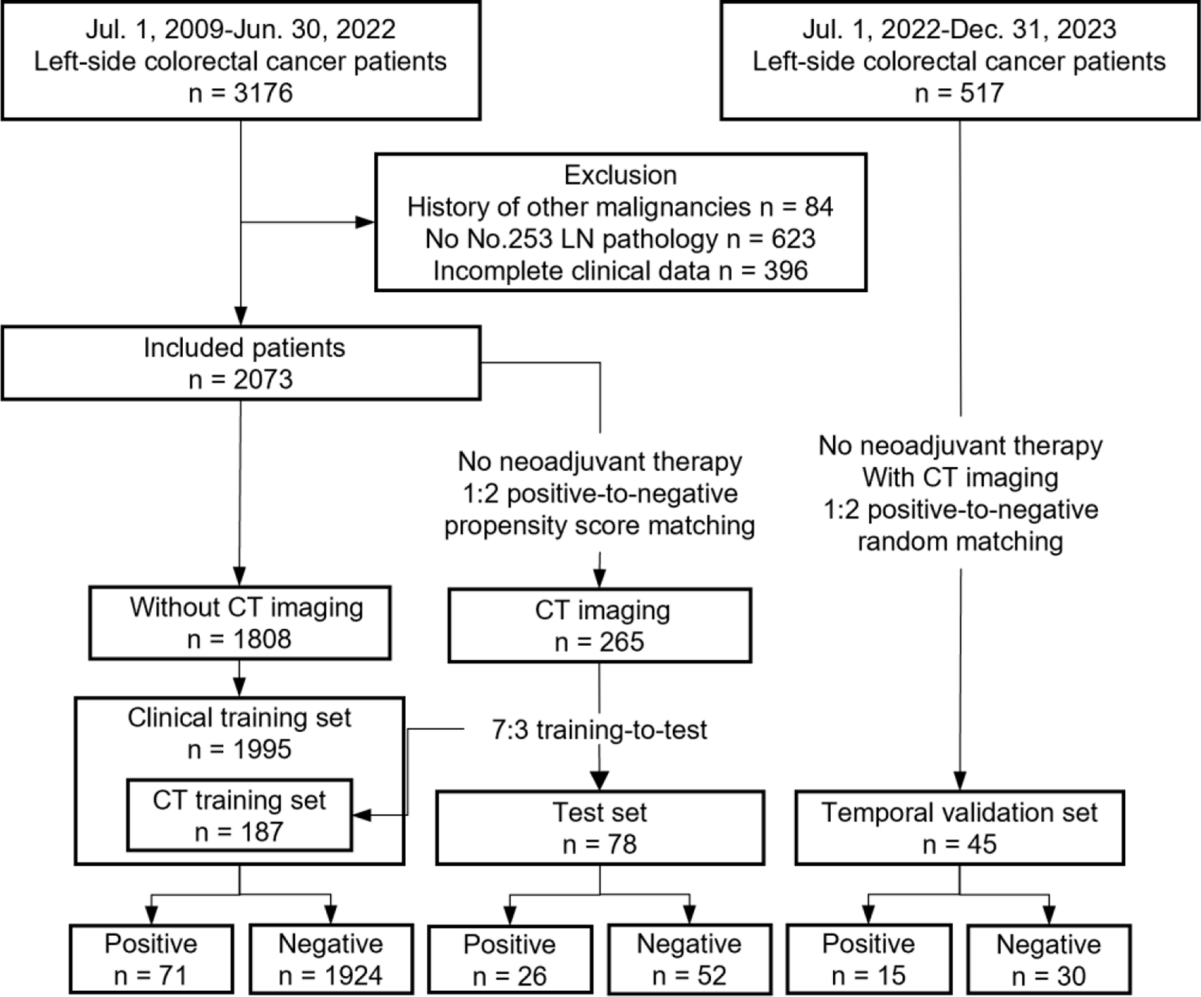
Study flow diagram. LN = lymph node

After applying the inclusion and exclusion criteria, this study ultimately collected clinical data from 2,118 patients and contrast-enhanced CT images from 310 of these patients. The training set included clinical data from 1,995 patients and CT images from 187 patients. The testing set comprised 78 patients, and there was a temporal validation set of 45 patients (Fig. 3).

The main characteristics of the patients, surgeries, and pathological findings are summarized in Table 1. The average age of the patients was 64 years, with a slight male predominance, and nearly 70% were diagnosed with rectal cancer. The majority of patients were classified as stage T2-T3 and stage N0-N1. A total of 112 patients exhibited No. 253 LN metastasis. Additional clinical characteristics are detailed in Supplementary Table 1.

**Table 1:** Main patients, surgical and pathological characteristics

Characteristic	No. 253 LN negative *n* = 2006	No. 253 LN positive *n* = 112	*p*-value
Age, years	64.0 [56.0, 72.0]	63.0 [56.8, 70.2]	0.373
Sex, male	1263 (63.0%)	76 (67.9%)	0.345
CEA, high	735 (36.6%)	78 (69.6%)	< 0.001
CA19-9, high	219 (10.9%)	31 (27.7%)	< 0.001
CA24-2, high	419 (20.9%)	49 (43.8%)	< 0.001
Tumor site			< 0.001
Rectum	1388 (69.2%)	58 (51.8%)	
Sigmoid colon	529 (26.4%)	50 (44.6%)	
Descending colon	89 (4.44%)	4 (3.57%)	
Distance from anal verge, cm	10.0 [6.00, 19.0]	15.0 [10.0, 20.0]	< 0.001
Endoscopic obstruction, yes	390 (19.4%)	50 (44.6%)	< 0.001
Synchronous liver metastasis, yes	62 (3.09%)	10 (8.93%)	0.004
Neoadjuvant, yes	40 (1.99%)	5 (4.46%)	0.086
Intraoperative No. 253 LN enlargement, yes	38 (1.89%)	21 (18.8%)	< 0.001
LCA ligation level, high	522 (26.0%)	49 (43.8%)	< 0.001
Gross type			0.002
Ulcerative	1382 (68.9%)	93 (83.0%)	
Expanding/Infiltrative	624 (31.1%)	19 (16.9%)	
Tumor differentiation			< 0.001
Well/moderately	1782 (88.8%)	79 (70.5%)	
Poorly/undifferentiation	224 (11.2%)	33 (29.5%)	
Pathological classification			0.003
Adenocarcinoma/Mucinous adenocarcinoma	2000 (99.7%)	109 (97.3%)	
Signet ring adenocarcinoma/Undifferentiated carcinoma	6 (0.30%)	3 (2.68%)	
Neural invasion			< 0.001
Yes	398 (19.8%)	66 (58.9%)	
No	1543 (76.9%)	44 (39.3%)	
Not reported	65 (3.24%)	2 (1.79%)	
Lymphovascular invasion			< 0.001
Yes	254 (12.7%)	43 (38.4%)	
No	1001 (49.9%)	1 (0.89%)	
Not reported	751 (37.4%)	68 (60.7%)	
AJCC pT classification			< 0.001
T1	170 (8.47%)	2 (1.79%)	
T2	416 (20.7%)	3 (2.68%)	
T3	1291 (64.4%)	81 (72.3%)	
T4	129 (6.43%)	26 (23.2%)	
AJCC pN classification			< 0.001
N0	1162 (57.9%)	0 (0.00%)	
N1	578 (28.8%)	25 (22.3%)	
N2	266 (13.3%)	87 (77.7%)	

Values are presented as median [lower quartile, upper quartile] or n (%)

AJCC = American Joint Committee on Cancer; LN = lymph node; LCA = left colic artery

### Feature selection

All features were selected into a multivariable logistic regression model using univariate logistic regression and the AIC method. The clinical model included six features: tumor site, endoscopic obstruction, CEA levels, growth type, differentiation grade, and pathological classification. These pieces of information can be obtained from preoperative endoscopic exams. We excluded information that can only be obtained intraoperatively and postoperatively, such as intraoperative No.253 LN enlargement, neural invasion, lymphovascular invasion, pathological T stage, and pathological N stage. The CT model incorporated average CT value, SAD and LAD of the largest No. 253 LN. The radiomics model included one first-order feature: the maximum gray level intensity within the ROI; two GLSZM features: Large Area High Gray Level Emphasis (LAHGLE) and Small Area High Gray Level Emphasis (SAHGLE); and one shape feature: Surface Area to Volume ratio. The odds ratios and p-values for these features are summarized in Table 2.

**Table 2:** Odds ratios of the included feature

Characteristic	Odds ratio	CI lower	CI upper	*p*-value
Tumor site, sigmoid colon	2.237	1.375	3.607	0.001
Endoscopic obstruction, yes	3.390	2.079	5.481	< 0.001
CEA, high	5.080	3.011	8.974	< 0.001
Growth type, ulcerative	2.771	1.476	5.784	0.003
Tumor differentiation, poorly/undifferentiation	3.167	1.814	5.333	< 0.001
Pathological classification, Signet ring adenocarcinoma/Undifferentiated carcinoma	14.103	2.928	54.652	< 0.001
LLN average CT value, HU	13.900	1.027	285.638	0.013
LLN SAD, mm	41.105	6.938	299.895	< 0.001
LLN LAD, mm	20.467	2.25	226.301	0.010
Firstorder_Maximum	647.267	10.982	75783.38	0.005
GLSZM_LargeAreaHighGrayLevelEmphasis	8.20 × 10^9^	4502.913	7.88 × 10^18^	0.011
GLSZM_SmallAreaHighGrayLevelEmphasis	9.257	1.527	61.773	0.017
Shape_SurfaceVolumeRatio	0.014	0.001	0.118	< 0.001

LN = lymph node; LLN = largest lymph node; SAD = short-axis diameter; LAD = long-axis diameter; Firstorder_Maximum = the maximum gray level intensity within the region of interest; GLSZM = Gray Level Size Zone Matrix

### Model evaluation

After establishing the clinical model, CT model, and radiomics model, we constructed a combined model by integrating the clinical model, CT model, and radiomics model. Each individual model was set to a threshold corresponding to 90% sensitivity. The combined model was defined as positive if all three individual models were positive. To evaluate these four models, we maximized Youden’s index and then calculated the AUC of the ROC, as well as accuracy (ACC), sensitivity (SEN), specificity (SPE), positive predictive value (PPV), and negative predictive value (NPV) in the test set and temporal validation set (Table 3).

**Table 3:** Evaluation metrics of the models

Model	AUC	ACC	SEN	SPE	PPV	NPV
Test set						
Clinical model	0.694	0.654	0.808	0.577	0.488	0.857
CT model	0.663	0.705	0.462	0.827	0.571	0.754
Radiomics model	0.720	0.667	0.731	0.635	0.500	0.825
Combined model	0.663	0.590	0.885	0.442	0.442	0.885
**Temporal validation set**						
Clinical model	0.743	0.644	0.867	0.533	0.481	0.889
CT model	0.629	0.644	0.933	0.500	0.483	0.938
Radiomics model	0.716	0.756	0.667	0.800	0.625	0.828
Combined model	0.800	0.800	0.800	0.800	0.667	0.889

The model determines the optimal decision threshold by maximizing Youden’s Index

AUC = area under the curve; ACC = accuracy; SEN = sensitivity; SPE = specificity; PPV = positive predictive value; NPV = negative predictive value

Specifically, since the SAD and LAD of lymph nodes are clinically used as indicators for assessing lymph node metastasis, we separately evaluated these two parameters, and the results are summarized in the Supplementary Table 2. The optimal threshold for the SAD was 10.2 mm, while that for the LAD was 11.1 mm. The predictive ability of the SAD was better than that of the LAD, but both were less effective compared to the CT model.

### Model comparison

**Fig. 4:**
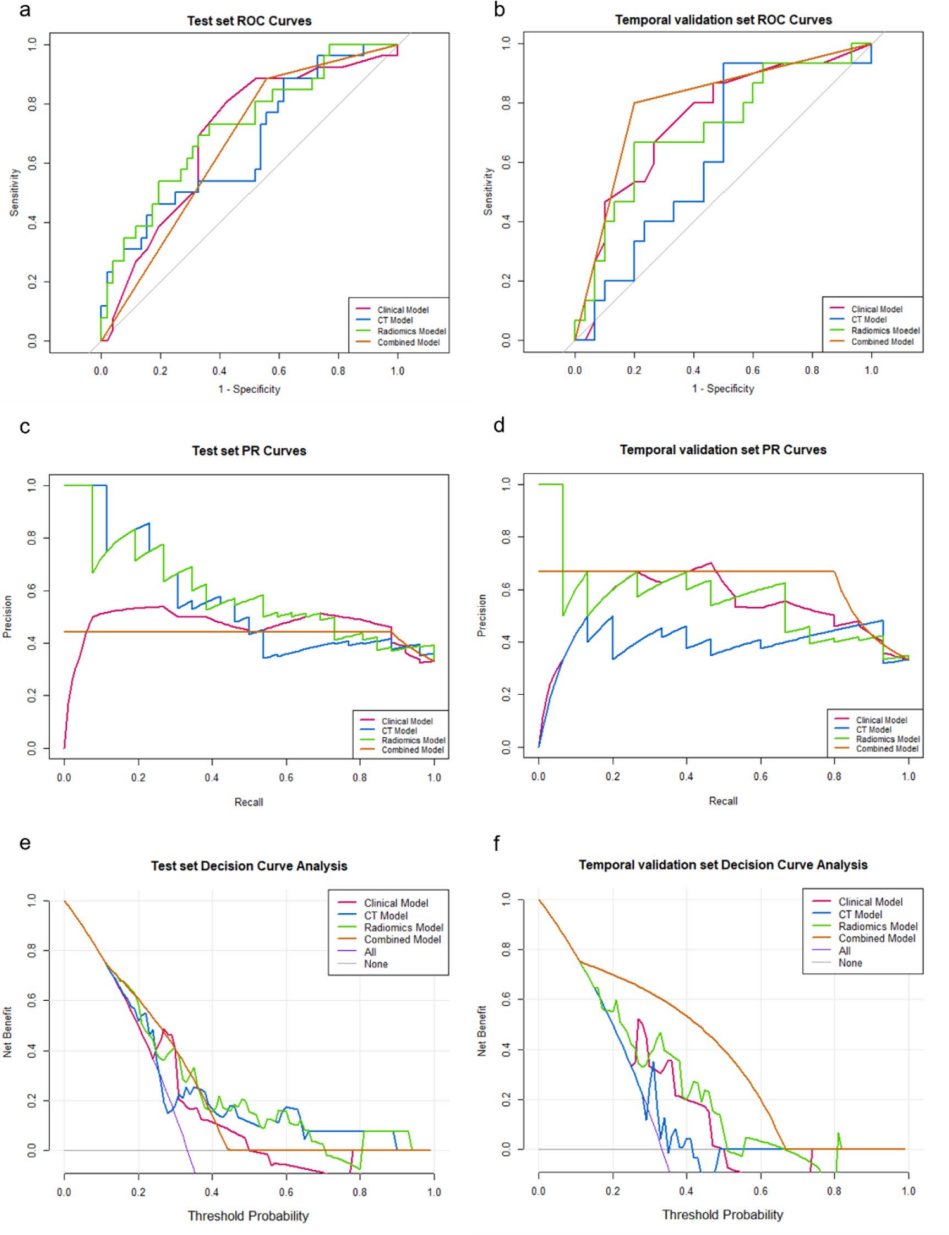
Comparison of the clinical, CT, radiomics, and combined models. ROC curves in the test set (**a**). ROC curves in the temporal validation set (**b**). PR curves in the test set (**c**). PR curves in the temporal validation set (**d**). DCA curves in the test set (**e**). DCA curves in the temporal validation set (**f**). ROC = receiver operating characteristic; PR = precision recall

Figure 4a and b show the ROC curves for the four models in the test set and temporal validation set, with the combined model demonstrating a better performance. Besides, the radiomics model slightly outperformed the CT model in both the test set and the temporal validation set. The clinical model performed well in the temporal validation set, benefiting from a large-scale clinical training set (*n* = 1995). However, it exhibited poorer performance in the test set due to the clinical data of the test set patients being adjusted through propensity matching. Additionally, we plotted the PR curves for the four models (Fig. 4c and d) and the DCA curves (Fig. 4e and f). Consistent with the results of the ROC curve, both the combined model and the clinical model performed well in the temporal validation set, while the radiomics model outperformed the CT model. Moreover, the model comparison graphs for SAD and LAD can be found in Supplementary Fig. 1. The predictive performance of using SAD or LAD alone is not satisfactory.

Supplementary Table 3 shows the relationship between the predictive probabilities of the combined model and the other three models, as well as the percentage of reclassification using the combined model. A comparison of the combined model with the clinical model, CT model, and radiomics model revealed that combined model can improve other models in temporal validation set.

## Discussion

No. 253 LN are the main lymph nodes for left-sided CRC, with a metastasis rate of around 6% [9, 18, 19]. In this study, the metastasis rate was found to be 5%. High ligation of IMA or dissection of No. 253 LN may injure the superior hypogastric plexus, leading to impairment of urogenital function [5]. In contract, low ligation of IMA better preserves urogenital function [6]. Besides, low ligation of IMA maintains the LCA, thereby improving blood supply to the proximal anastomosis and reducing anastomotic leakage occurrence [20]. The most contentious issue regarding low IMA ligation is the risk of residual tumor in No. 253 LN. Given the low incidence of metastasis in No. 253 LN, performing comprehensive lymph node dissection for all patients is unreasonable. Our study has developed a predictive model for No. 253 LN metastasis, significantly minimizing unnecessary lymph node dissection and reducing the oncological risk associated with low ligation of IMA.

Previous studies based on clinical information have identified predictive factors for metastasis in No. 253 LN, such as neoadjuvant, age, synchronous liver metastasis, synchronous lung metastasis, signet ring adenocarcinoma, neural invasion, lymphovascular invasion, CA199, endoscopic obstruction, and T stage evaluated by MRI [10]. However, it is challenging to accurately assess nerve invasion and lymphovascular invasion through preoperative colonoscopy pathology. Additionally, while MRI is routinely performed in patients with low rectal cancer, it is rarely conducted in patients with other types of colorectal cancer, which introduces certain limitations to previous studies.

Our study identified tumor site, endoscopic obstruction, CEA levels, growth type, differentiation grade, and pathological classification. Tumors located in the sigmoid colon are more likely to metastasize to No. 253 LN, possibly due to alternative lymphatic drainage pathways in the rectum and left colon. Endoscopic obstruction may be linked to increased intertumoral pressure, which promotes lymph node metastasis. CEA, a commonly used tumor marker, is associated with tumor burden. The tumor’s growth type, differentiation grade, and pathological classification reflect its malignancy, with highly malignant tumors being more prone to lymph node metastasis. Our clinical information-based predictive model achieved an AUC of 0.743 in temporal validation set. In addition to the features included in the model, other tumor markers, neural invasion, lymphovascular invasion, and lymphocyte response within the tumor are also associated with metastasis in No. 253 LN.

In recent years, radiomics has rapidly evolved, enabling high-throughput extraction of patterns from medical images that are invisible to the naked eye [15, 21]. Traditionally, radiologists have assessed lymph node metastasis based on SAD, heterogeneous enhancement and cystic degeneration, often with low accuracy [8]. Our study found that the optimal threshold for SAD is 10.2 mm. Compared to LAD, SAD serves as a simpler and more effective diagnostic indicator, which aligns with clinical experience. Some studies have used images of the primary tumor to predict the N stage in colorectal cancer patients, aiding in the selection of neoadjuvant treatment [22]. The location of No. 253 LN is well-defined, discernible via IMA, LCA, and IMV, making it easy to locate and annotate on CT images. Contrast-enhanced CT is a routine examination tool for CRC, offering high generalizability for CT-based predictive models. Therefore, it is very promising to establish a CT-based radiomics model for predicting No. 253 LN metastasis.

Our study identified the largest lymph node average CT value, SAD and LAD as predictive factors for the CT model, which aligns with the expertise of radiologists. However, models based solely on CT features exhibit poor predictive performance, with an AUC of 0.663 in test set and 0.629 in temporal validation set, indicating the limitations of visual assessment. In radiomics models, one first-order feature, two GLSZM features, and one shape feature were closely related to metastasis in No. 253 LN. The maximum gray level intensity within the ROI reflects the abnormal enhancement of tumor tissue on contrast-enhanced CT scans. Changes in GLSZM features might reflect differences between metastatic tumors and surrounding normal tissue. The surface area to volume ratio reflects the tendency of metastatic lymph nodes to become more irregular due to tumor infiltration. What’s more, some studies have shown that radiomics can predict tumor chemotherapy responsiveness, revealing that molecular changes might manifest macroscopically and be detectable through radiomics [23, 24].

Our study developed a clinical, a CT, a radiomics, and a combined model. In the temporal validation set, the clinical model demonstrated robust performance, attributable to the substantial sample size. Models based solely on CT or radiomics features showed moderate performance, with AUCs of 0.629 and 0.716, respectively. The combined model performed best, achieving an AUC of 0.8 and showing moderate improvements in IDI and INR compared to other models. This highlights the tremendous potential of combining clinical characteristics with radiomics features in predicting lymph node metastasis.

Our study has several limitations that should be acknowledged. First, this is a single-center retrospective study with temporal validation only, lacking external spatial validation, which may limit the generalizability of our findings. Second, although the No. 253 region is relatively small and the correspondence between imaging and pathology is relatively good, achieving a one-to-one correspondence remains challenging. This limitation may have restricted the performance of both the CT model and the radiomics model. Third, due to the significant disparity in the data scale between clinical and imaging datasets, we employed propensity score matching to construct the imaging cohort and used logical rules to build the combined model. However, this approach may not have fully leveraged the potential of the CT and radiomics models. Future studies should aim to expand the imaging dataset to address this issue. Lastly, while we utilized interpretable logistic regression models and logical rules in this study, more complex models, such as neural networks and deep learning, could be applied once the imaging dataset is expanded, potentially yielding better results.

## Conclusion

A predictive model incorporating clinical and CT-based radiomics features demonstrates the potential of radiomics in predicting No. 253 LN metastasis in patients with left-sided CRC. Moreover, this model provides valuable insights for guiding the optimal IMA ligation strategy.

## Electronic supplementary material

Below is the link to the electronic supplementary material.

Supplementary Material 1

## Data Availability

The datasets used and/or analyzed during the current study are available from the corresponding author on reasonable request.

## Abbreviations

CRC: Colorectal Cancer
IMA: Inferior Mesenteric Artery
No. 253 LN: No. 253 Lymph Node
LCA: Left Colic Artery
CT: Computed Tomography
MRI: Magnetic Resonance Imaging
AUC: Area Under the Curve
IMV: Inferior Mesentery Vein
SAD: Short-Axis Diameter
LAD: Long-Axis Diameter
GLCM: Gray-Level Co-Occurrence Matrix
GLDM: Gray-Level Dependence Matrix
GLRLM: Gray-Level Run Length Matrix
GLSZM: Gray-Level Size Zone Matrix
AIC: Akaike Information Criterion
VIF: Variance Inflation Factor
PCC: Pearson Correlation Coefficient
ROC: Receiver Operating Characteristic
PR: Precision–Recall
DCA: Decision Curve Analysis
NRI: Net Reclassification Improvement
IDI: Integrated Discrimination Improvement
LAHGLE: Large Area High Gray Level Emphasis
ZV: Zone Variance
ACC: Accuracy
SEN: Sensitivity
SPE: Specificity
PPV: Positive Predictive Value
NPV: Negative Predictive Value
