# Supplementary material for: Development and validation of machine learning models for predicting no. 253 lymph node metastasis in left-sided colorectal cancer using clinical and CT-based radiomic features
Source: Cancer Imaging. 2025 Apr 29;25:57. doi: 10.1186/s40644-025-00876-y (PMC12039209; doi:10.1186/s40644-025-00876-y)
Supplement: Supplementary file 1 — Supplementary Material 1 [file 40644_2025_876_MOESM1_ESM.docx]

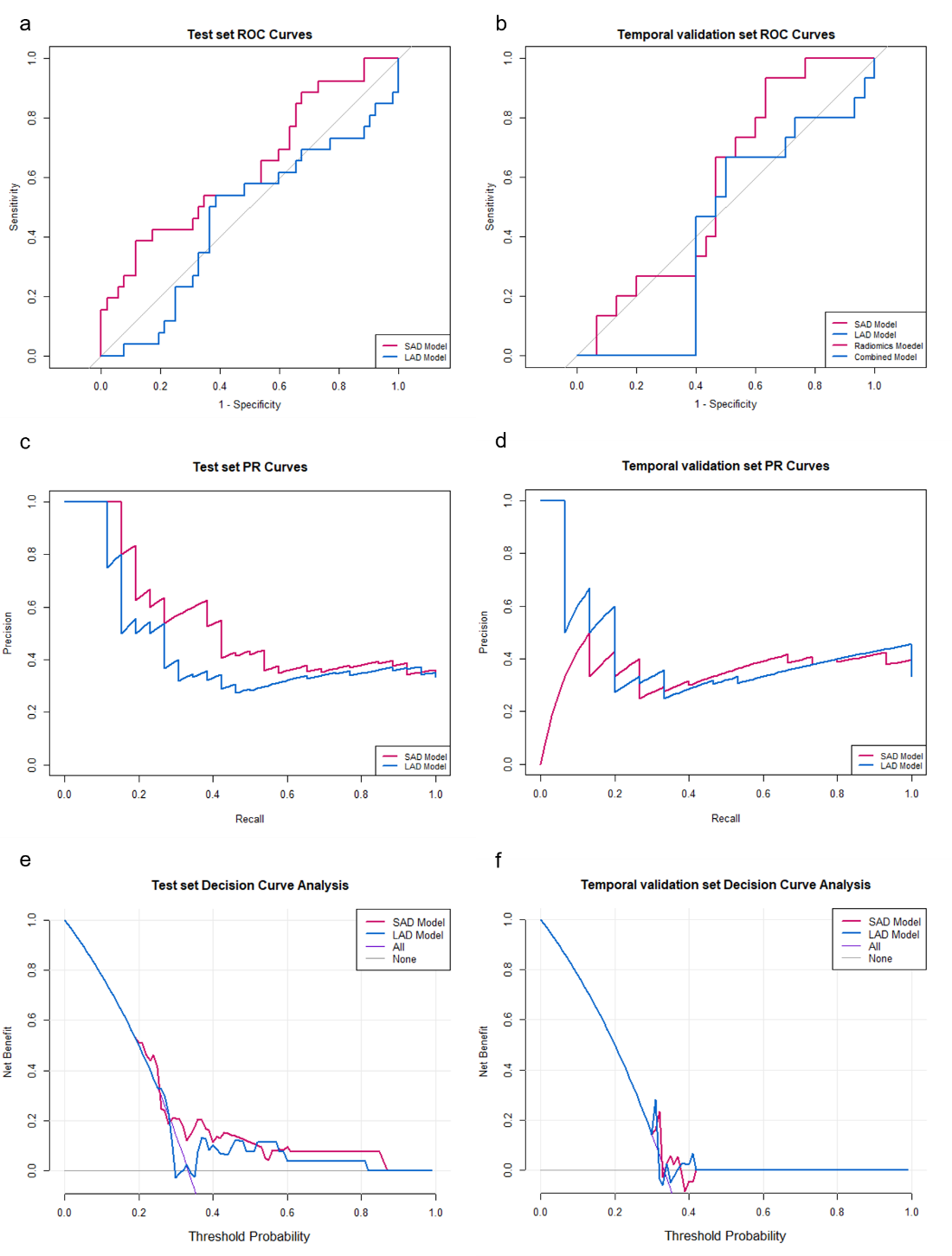
**Supplementary Figure.1** Comparison of the SAD and LAD models. ROC curves in the test set (a). ROC curves in the temporal validation set (b). PR curves in the test set (c). PR curves in the temporal validation set (d). DCA curves in the test set (e). DCA curves in the temporal validation set (f).

SAD = short-axis diameter; LAD = long-axis diameter; ROC = receiver operating characteristic; PR = precision recall.

**Supplementary Table 1** Other patients, surgical and pathological characteristics

| **Characteristic** | **No. 253 LN negative**  **n = 2006** | **No. 253 LN positive**  **n = 112** | **p-value** |
| --- | --- | --- | --- |
| Height, cm | 167 [160, 172] | 170 [162, 173] | 0.041 |
| Weight, kg | 66.0 [60.0, 75.0] | 68.5 [58.8, 75.0] | 0.671 |
| BMI, kg/m^2^ | 24.1 [21.9, 26.1] | 24.1 [21.1, 25.8] | 0.266 |
| Hypertension, yes | 768 (38.3%) | 41 (36.6%) | 0.798 |
| Diabetes, yes | 325 (16.2%) | 23 (20.5%) | 0.283 |
| Coronary heart disease, yes | 204 (10.2%) | 12 (10.7%) | 0.980 |
| Heart failure, yes | 13 (0.65%) | 0 (0.00%) | 1.000 |
| Cerebral infarction, yes | 149 (7.43%) | 7 (6.25%) | 0.781 |
| Renal insufficiency, yes | 11 (0.55%) | 0 (0.00%) | 1.000 |
| Liver cirrhosis, yes | 6 (0.30%) | 0 (0.00%) | 1.000 |
| CA72-4, high | 529 (26.4%) | 35 (31.3%) | 0.304 |
| Maximum tumor diameter, cm | 4.00 [3.00, 5.00] | 4.00 [3.50, 5.50] | 0.147 |
| Endoscopic therapy, yes | 20 (1.00%) | 0 (0.00%) | 0.622 |
| Synchronous pulmonary metastasis, yes | 23 (1.15%) | 1 (0.89%) | 1.000 |
| Synchronous peritoneal metastasis, yes | 0 (0.00%) | 1 (0.89%) | 0.053 |
| Multiple Primary Colorectal Cancer, yes | 50 (2.49%) | 0 (0.00%) | 0.109 |
| ASA classification |  |  | 0.231 |
| 1 | 108 (5.38%) | 10 (8.93%) |  |
| 2 | 1441 (71.8%) | 73 (65.2%) |  |
| 3 | 442 (22.0%) | 28 (25.0%) |  |
| 4 | 15 (0.75%) | 1 (0.89%) |  |
| Surgery type |  |  | 1.000 |
| Laparoscopic | 1519 (75.7%) | 85 (75.9%) |  |
| Open | 487 (24.3%) | 27 (24.1%) |  |
| Surgical procedure |  |  | 0.002 |
| Dixon | 1605 (80.0%) | 87 (77.7%) |  |
| Miles | 223 (11.1%) | 8 (7.14%) |  |
| Left hemicolectomy | 132 (6.58%) | 9 (8.04%) |  |
| Hartmann | 21 (1.05%) | 8 (7.14%) |  |
| Total colectomy | 14 (0.70%) | 0 (0.00%) |  |
| Total pelvic exenteration | 10 (0.50%) | 0 (0.00%) |  |
| Intratumor lymphocyte response |  |  | 0.003 |
| Mild | 1146 (57.1%) | 84 (75.0%) |  |
| Moderate | 650 (32.4%) | 21 (18.8%) |  |
| Severe | 77 (3.84%) | 3 (2.68%) |  |
| Not reported | 133 (6.63%) | 4 (3.57%) |  |
| Peritumor lymphocyte response |  |  | 0.001 |
| Mild | 1012 (50.4%) | 78 (69.6%) |  |
| Moderate | 766 (38.2%) | 26 (23.2%) |  |
| Severe | 111 (5.53%) | 4 (3.57%) |  |
| Not reported | 117 (5.83%) | 4 (3.57%) |  |
| Resection margins |  |  | 0.016 |
| R0 | 1982 (98.8%) | 107 (95.5%) |  |
| R1 | 24 (1.20%) | 5 (4.46%) |  |

Values are presented as median [lower quartile, upper quartile] or n (%).

LN = lymph node.

**Supplementary Table 2** Evaluation metrics of the SAD and LAD models.

| **Model** | **AUC** | **ACC** | **SEN** | **SPE** | **PPV** | **NPV** |
| --- | --- | --- | --- | --- | --- | --- |
| Test set |  |  |  |  |  |  |
| SAD model | 0.632 | 0.718 | 0.385 | 0.885 | 0.625 | 0.742 |
| LAD model | 0.465 | 0.410 | 0.462 | 0.385 | 0.273 | 0.588 |
| Temporal validation set |  |  |  |  |  |  |
| SAD model | 0.578 | 0.556 | 0.933 | 0.367 | 0.424 | 0.917 |
| LAD model | 0.427 | 0.444 | 0.333 | 0.500 | 0.250 | 0.600 |

The model determines the optimal decision threshold by maximizing Youden's Index.

SAD = short-axis diameter; LAD = long-axis diameter; AUC = area under the curve; ACC = accuracy; SEN = sensitivity; SPE = specificity; PPV = positive predictive value; NPV = negative predictive value.

**Supplementary Table 3** NRI and IDI of the combined model related to the other models.

|  | | **Combined model** | | | **NRI** | **IDI** |
| --- | --- | --- | --- | --- | --- | --- |
|  |  | [0, 0.5) | [0.5, 1] | Reclassified (n%) |  |  |
| **Test set** | | | | | | |
| Clinical model | [0, 0.0379) | 16 | 20 | 43 | -0.0385 (-0.2376 -0.1607, p = 0.705) | 0.2982 (0.1145 -0.4819, p = 0.001) |
|  | [0.0379, 1] | 10 | 40 | 20 |  |  |
| CT model | [0, 0.3604) | 24 | 31 | 56 | 0.0769 (-0.1819 -0.3358, p = 0.560) | 0.2159 (0.0268 -0.4049, p = 0.025) |
|  | [0.3604, 1] | 2 | 21 | 9 |  |  |
| Radiomics model | [0, 0.3183) | 20 | 30 | 60 | 0.0577 (-0.2299 -0.3453, p = 0.694) | 0.1863 (-0.0073 -0.3800, p = 0.059) |
|  | [0.3183, 1] | 6 | 22 | 21 |  |  |
| **Temporal validation set** | | | | | | |
| Clinical model | [0, 0.0379) | 25 | 5 | 17 | 0.3000 (0.0495 -0.5505, p = 0.019) | 0.5770 (0.3357 -0.8114, p < 0.001) |
|  | [0.0379, 1] | 2 | 13 | 13 |  |  |
| CT model | [0, 0.3604) | 25 | 13 | 34 | 0.5333 (0.1985 -0.8682, p = 0.001) | 0.5722 (0.3182 -0.8262, p < 0.001) |
|  | [0.3604, 1] | 1 | 6 | 14 |  |  |
| Radiomics model | [0, 0.3183) | 19 | 4 | 17 | 0.3333 (0.0288 - 0.6379, p = 0.032) | 0.4383 (0.1782 -0.6985, p < 0.001) |
|  | [0.3183, 1] | 8 | 14 | 36 |  |  |

NRI = net reclassification improvement; IDI = integrated discrimination improvement.
